# Karyotypical characteristics of two allopatric African populations of anhydrobiotic *Polypedilum* Kieffer, 1912 (Diptera, Chironomidae) originating from Nigeria and Malawi

**DOI:** 10.3897/CompCytogen.v9i2.9104

**Published:** 2015-05-11

**Authors:** Ninel A. Petrova, Richard Cornette, Sachiko Shimura, Oleg A. Gusev, Dylo Pemba, Takahiro Kikawada, Sergey V. Zhirov, Takashi Okuda

**Affiliations:** 1 Zoological Institute, Russian Academy of Sciences, Universitetskaya nab. 1, St. Petersburg 199034, Russia; 2 National Institute of Agrobiological Sciences, 1-2 Ohwashi, Tsukuba, Ibaraki 305-8634, Japan; 3 Institute of Fundamental Biology and Medicine, Kazan Federal University, Kremlevskaya ul., 18, Kazan, 420008 Russia; 4 Biological Sciences Department Chancellor college, University of Malawi, P.O.Box 280, Zomba, Malawi

**Keywords:** Chironomidae, *Polypedilum vanderplanki*, allopatric populations, Nigeria, Malawi, anhydrobiosis, polytene chromosomes

## Abstract

The African chironomid *Polypedilum vanderplanki* Hinton, 1951 is the only chironomid able to withstand almost complete desiccation in an ametabolic state known as anhydrobiosis. The karyotypes of two allopatric populations of this anhydrobiotic chironomid, one from Nigeria and another from Malawi, were described according to the polytene giant chromosomes. The karyotype from the Nigerian population was presented as the reference chromosome map for *Polypedilum vanderplanki*. Both populations, Nigerian and Malawian, showed the same number of chromosomes (2n=8), but important differences were found in the band sequences of polytene chromosomes, and in the number and the arrangement of active regions between the two populations. Such important differences raise the possibility that the Malawian population could constitute a distinct new species of anhydrobiotic chironomid.

## Introduction

The African non-biting midge, *Polypedilum vanderplanki* Hinton, 1951, is the only species among the family Chironomidae and also among all insects showing tolerance to almost complete dehydration, although another *Polypedilum* species was also suggested to exhibit similar desiccation tolerance (Hinton 1951, 1960a,b, Watanabe 2006, Cranston 2014). Larvae of this species live in small rock pools on granite outcrops in the semi-arid regions of Africa. During the dry season, water evaporates rapidly and the larvae are capable of surviving in a quiescent desiccated state in dry mud for several months. After rain falls, the dried larvae rehydrate rapidly, returning to active life, and resume normal development. Such an ability to survive severe desiccation in an ametabolic quiescent state is known as anhydrobiosis and the sleeping chironomid is the largest anhydrobiotic animal known to date (Watanabe 2006).

During the last decade, extensive physiological and molecular studies were performed to understand the mechanisms underlying anhydrobiosis in *Polypedilum vanderplanki* larvae (Cornette et al. 2010, Cornette and Kikawada 2011, Watanabe et al. 2002). During the desiccation process, larvae accumulate a large amount of trehalose, a nonreducing sugar that replaces water in cells and eventually forms a glassy matrix protecting biological molecules during anhydrobiosis (Watanabe et al. 2002, Watanabe et al. 2003, Kikawada et al. 2007, Sakurai et al. 2008). During anhydrobiosis, protective proteins such as heat shock proteins or late embryogenesis abundant proteins are also abundantly produced in order to prevent protein aggregation due to desiccation (Kikawada et al. 2006, Gusev et al. 2011, Hatanaka et al. 2013). In addition, *Polypedilum vanderplanki* larvae have to face oxidative stress during anhydrobiosis and express a large array of antioxidants (Cornette et al. 2010, Gusev et al. 2010). However, nuclear DNA experiences severe damage just after rehydration of dry larvae and DNA repair occurs slowly during the few days following rehydration (Gusev et al. 2010). Recently, comparative genome analysis showed that the genome of *Polypedilum vanderplanki* presents specific islands containing clusters of genes involved in anhydrobiosis (Gusev et al. 2014).

Taken together, all these data make *Polypedilum vanderplanki* an important model to study the phenomenon of anhydrobiosis in animals. Furthermore, this chironomid species was subjected to several studies with gamma- and ion beam irradiation, and the high radiotolerance of *Polypedilum vanderplanki* is now well characterized (Watanabe et al. 2005). As a consequence, *Polypedilum vanderplanki* was selected for several space experiments onboard and outside the International Space Station, including long-term exposure. This African chironomid is now recognized as an important anhydrobiotic model in the field of astrobiology. All these studies on anhydrobiosis, radiotolerance and exposure to space environment imply DNA damage, repair and possible chromosomal rearrangements. However, no cytogenetic data were available for *Polypedilum vanderplanki* to check the aforementioned effects on nuclear DNA. In addition, sequencing of the genome of *Polypedilum vanderplanki* raised the need for a chromosomal description in order to establish a physical map of the genome data.

Thus, the present study reports a detailed description of polytene chromosomes of *Polypedilum vanderplanki* larvae. The goal of this work was to establish a reference map of the *Polypedilum vanderplanki* karyotype and to estimate the cytological differences between two distant populations.

Anhydrobiotic chironomid larvae from two distant African populations originating from Nigeria and Malawi were investigated. The reference karyotype for *Polypedilum vanderplanki* was obtained from the Nigerian population. Analysis of the polytene chromosomes showed that the diploid number of chromosomes (2n=8) and their ratio were identical in both populations. However, considerable cytogenetic differences were observed between both populations in the band sequences of polytene chromosomes, and in the number and the arrangement of active regions. The results of our research raise the possibility that the Malawian population may constitute a distinct new species of anhydrobiotic chironomids from Africa.

## Material and methods

Chironomids were reared in the laboratory at NIAS (Japan) as described in Watanabe et al. (2002). Briefly, chironomid larvae were reared on milk-agar under a 13:11h light:dark photoregime at 27–28 °C. Anhydrobiosis was induced by slow desiccation of the larvae as described previously (Watanabe et al. 2002, Kikawada et al. 2005).

The Nigerian strain of *Polypedilum vanderplanki* kept in the laboratory was an inbred line originating from different populations collected in small rock pools on granite outcrops around Zaria, close to the original collection points of the *Polypedilum vanderplanki* type specimen as described by Hinton (1951). Larvae from the Malawian population were collected in similar rock pools on granite outcrops in the vicinity of Mandala (14°06'044S, 33°59'517E) about 30 km South East from Lilongwe. Larvae were artificially desiccated and stored in desiccators at <5% relative humidity. Under such conditions, anhydrobiotic larvae remain viable for several years.

Larvae from both strains were placed in water at room temperature to rehydrate. Within a few hours, larvae were able to move and eat, i.e. came back to usual way of existence. The recovered larvae were maintained for 6–7 days, fed with a hay meal. After maintaining some of the larvae in good condition, they grew up and were ready to be used in the preparation of karyological slides.

Larvae were fixed in Clark’s liquid: 96% of ethyl alcohol and glacial acetic acid (3:1). Fixed material was stored at low temperature (4–6 °C). Twenty six larvae from the Nigerian population and 28 from the Malawian population were suitable for preparations.

For the preparation of the karyological slides of the polytene chromosomes, dissected salivary glands were stained in a 2% solution of acetoorcein. After short maceration into 50% lactic acid, the giant cells were separated from the secretion. Squash preparations were made following the routine method described previously (Chubareva and Petrova 1982). The photographs of the chromosomes were made at the magnification 100×.

The cytophotomaps of the polytene chromosomes from *Polypedilum vanderplanki* are published for the first time. Cytophotomaps were obtained for the representatives of both Nigerian and Malawian populations. Division of the chromosomes into sections was performed arbitrarily. Arms of the chromosomes were designated: I – AB, II – CD, III – EF, IV – G, according to the standard accepted for *Polypedilum nubifer* by Porter and Martin (1977), which was inferred from the system of nomenclature for Chironomidae (Keyl, 1960). This system of nomenclature does not imply homology with the arms A to G in the genus *Chironomus* (Porter & Martin, 1977).

## Results and discussion

### Karyotype of larvae from Nigeria

Salivary glands consisted of 16–20 cells. On the anterior end of the gland, there were 4 especially large cells, which contained the supergiant chromosomes. They were characterized by a high degree of polytenization and with clear morphology of bands. The best sample was selected for mapping. In other salivary gland cells, polytene chromosomes formed meandric breaks and did not show a perfectly clear picture of the bands.

**Figure 1.**
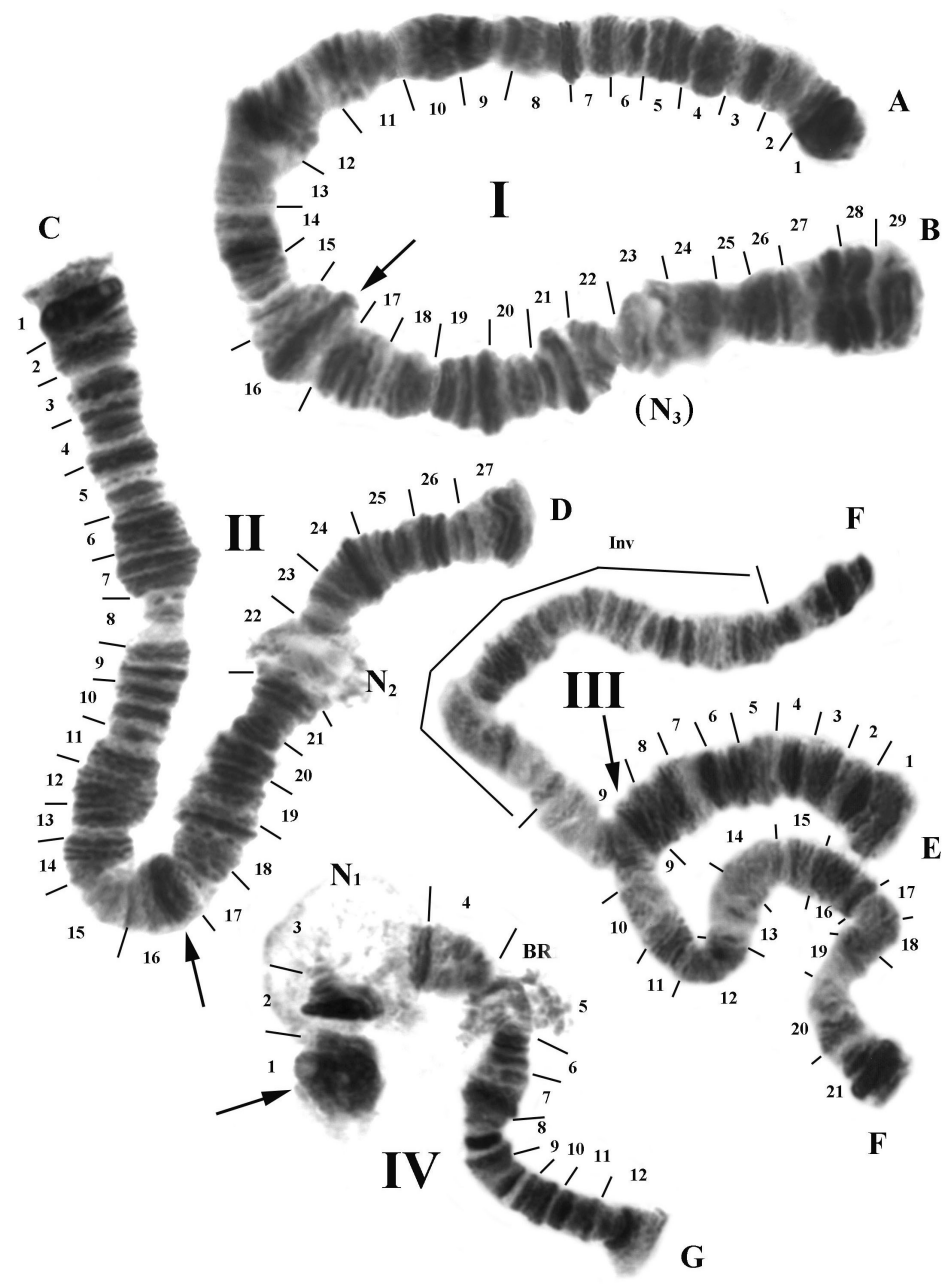
Representative karyotype of the *Polypedilum vanderplanki* population from Nigeria. Chromosome numbers are indicated as **I, II, III** and **IV.** Chromosome arms are labeled **A–B, C–D, E–F**, and **G.** The expected locations of the centromeres are indicated by arrows and each section is numbered and delimited by short lines. N_1_, N_2_, (N_3_): nucleoli, BR: Balbiani ring, Inv: inversion.

The diploid chromosome number coincided with the modal diploid number of the genus *Polypedilum*: 2n=8 (Fig. 1) (Tavcar 1967, Porter and Martin 1977, Petrova et al. 1981, Kiknadze et al. 1991, Gavrikova and Belyanina 1993, Kerkis et al. 1996, Michailova 1989). Chromosomes were designated according to their respective lengths - I, II, III and IV, with the length ratio I=II>III>IV. The combination of arms in chromosomes was AB, CD, EF and G (Keyl 1960). It should be noted that these designations do not mean a homology with arms in the genus *Chironomus*. Chromosome I was metacentric, chromosomes II and III submetacentrics, and chromosome IV telocentric. The putative centromeres were clearly visible (specified by arrows in Figure 1) and looked like conspicuous heterochromatic bands, wider than the average width of the chromosome. The karyotype of the population is multinucleolar, with two nucleoli (N).

**Figure 2.**
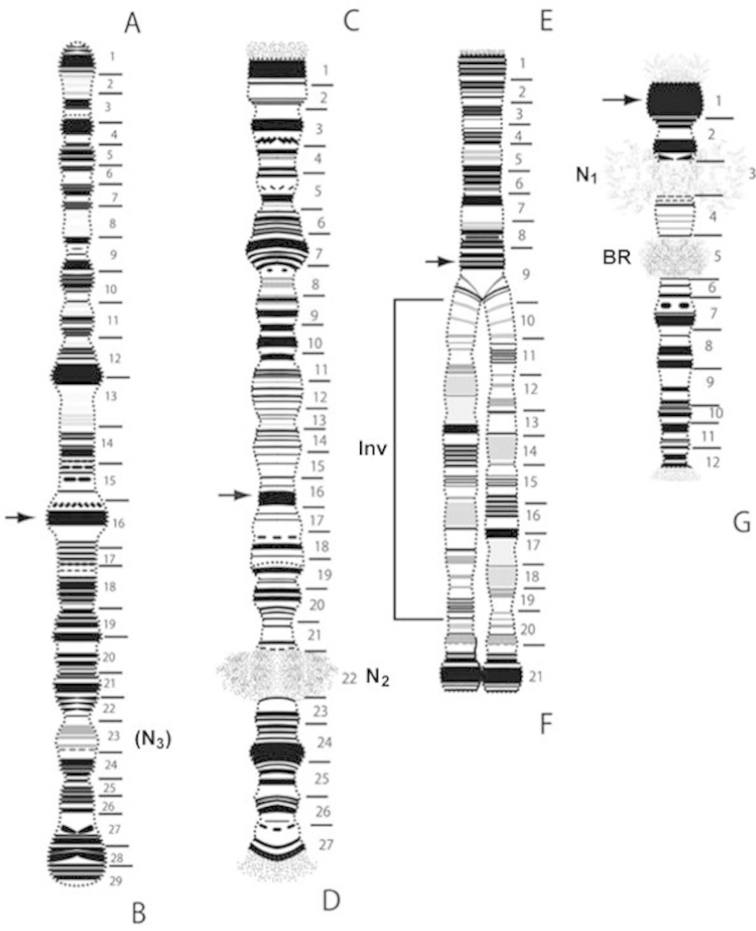
A simplified reference map for the *Polypedilum vanderplanki* Nigerian population. Chromosomes **I, II, III** and **IV** are shown in order from the left to the right. Numbering and abbreviations as described in Figure 1.

A simplified reference map of the *Polypedilum vanderplanki* lab strain is presented in Fig. 2.

Chromosome I was arbitrarily divided into 29 sections. The putative centromere was localized in sec. 16. The puff located in sec. 23 looked like a facultative nucleolus (designated (N_3_) in Figs 1 and 2). This puff was not observed in all cells.

**Figure 3.**
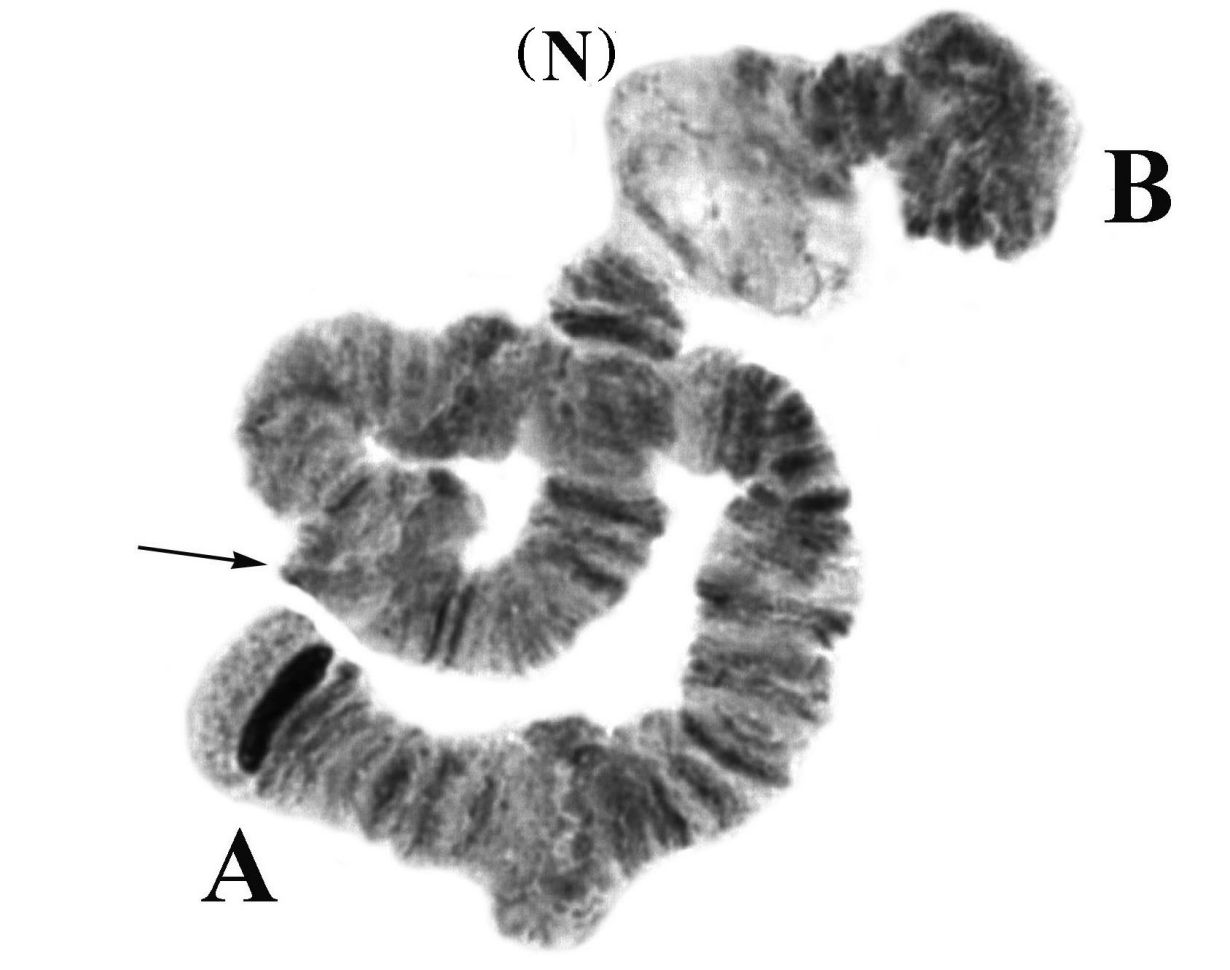
Chromosome I from the Nigerian *Polypedilum vanderplanki* population. Chromosome arms are labeled **A** and **B.** Arrow: location of the putative centromere, (N): activated (N_3_) nucleolus.

However, in some supergiant cells, this puff was in an active state and looked as a normal large nucleolus (designated (N_3_) in Fig. 3).

The marker for arm A was the dark block consisting of composite bands near the telomere. In addition, the groups of bands in sec. 4–5 and 9 can also serve as markers of this arm. Narrowing of chromosome width was observed in sec. 7 and 9. The arm B may easily be distinguished by a narrowing on the border of sec. 18–19 and by three thick heterochromatic bands almost identical near this narrowing in sec. 19. The next narrowing was conspicuous and observed on the border of sec. 22 and 23, before the facultative nucleolus N_3_. A wide dark heterochromatic block, consisting of 5 composite bands, was located at the telomere of arm B (Figs 1, 2).

Chromosome II was divided into 27 sections. The putative centromere was apparently localized in sec. 16. Markers for arm C were the wide dark heterochromatic block in sec. 1 near the telomere, and the narrowing in sec. 8, which was bordered with easily recognizable groups of bands in sec. 6–7 and in sec. 9–10. In the arm D, the main nucleolus N_2_ (sec. 22) was active and constantly present in all cells. The large dark block in sec.24 and groups of conspicuous bands in sec. 18, 20–21 and 26 constituted good markers for this arm (Figs 1, 2).

Chromosome III was divided into 21 sections (Figs 1, 2). The putative centromere was apparently in sec. 9. In all studied individuals, this chromosome presented only homologues of arm E conjugated, whereas in arm F the homologues were constantly uncoupled, due to a large inversion in sec. 11–19. Sometimes, sites near the telomere of arm F did conjugate, owing to an ectopic attraction of large heterochromatic blocks. Uncoupled homologues formed a large number of the meandric breaks and torsions. Arm E was well distinguished on evenly repeating groups of bands in sec. 2–4, 6–7, just as observed in the arm F in species of the genus *Chironomus* (Panis et al. 1994). In arm F, the main marker was the conjoint heterochromatic block in sec. 21, near the telomere. However, groups of bands in sec. 11 and 16 were also easily identified.

Chromosome IV was divided into 12 sections (Figs 1, 2). The putative centromere was localized in sec. 1. The nucleolus N_1_ was well developed and localized in chromosome IV, near the centromere (sec. 3). The morphology of this chromosome was slightly different from the other elements of the karyotype: it was half the thickness of any of the long chromosomes. It is possible to assume that the homologues of the chromosome IV are characterized by a lower degree of polytenization, compared to other chromosomes. Such a phenomenon has been observed in some species from the subfamily Chironominae (genus *Sergentia*) and Diamesinae (genus *Sympotthastia*) (Kerkis 1992, Proviz and Proviz 1992). Despite the prevalence of this phenomenon, it is impossible to be certain of the reason for its appearance. Next markers were the accurate dark bands located from both sides of N_1_ and a well developed Balbiani ring (BR) in sec. 5. Besides these markers, the band sequence in arm G formed an easily recognizable picture.

The population was inbred in the laboratory and this explains the low variability observed for chromosomal rearrangements. The only inversion – InvF (10–20) on chromosome III was found with a frequency of 100%.

### Karyotype of larvae from Malawi

**Figure 4.**
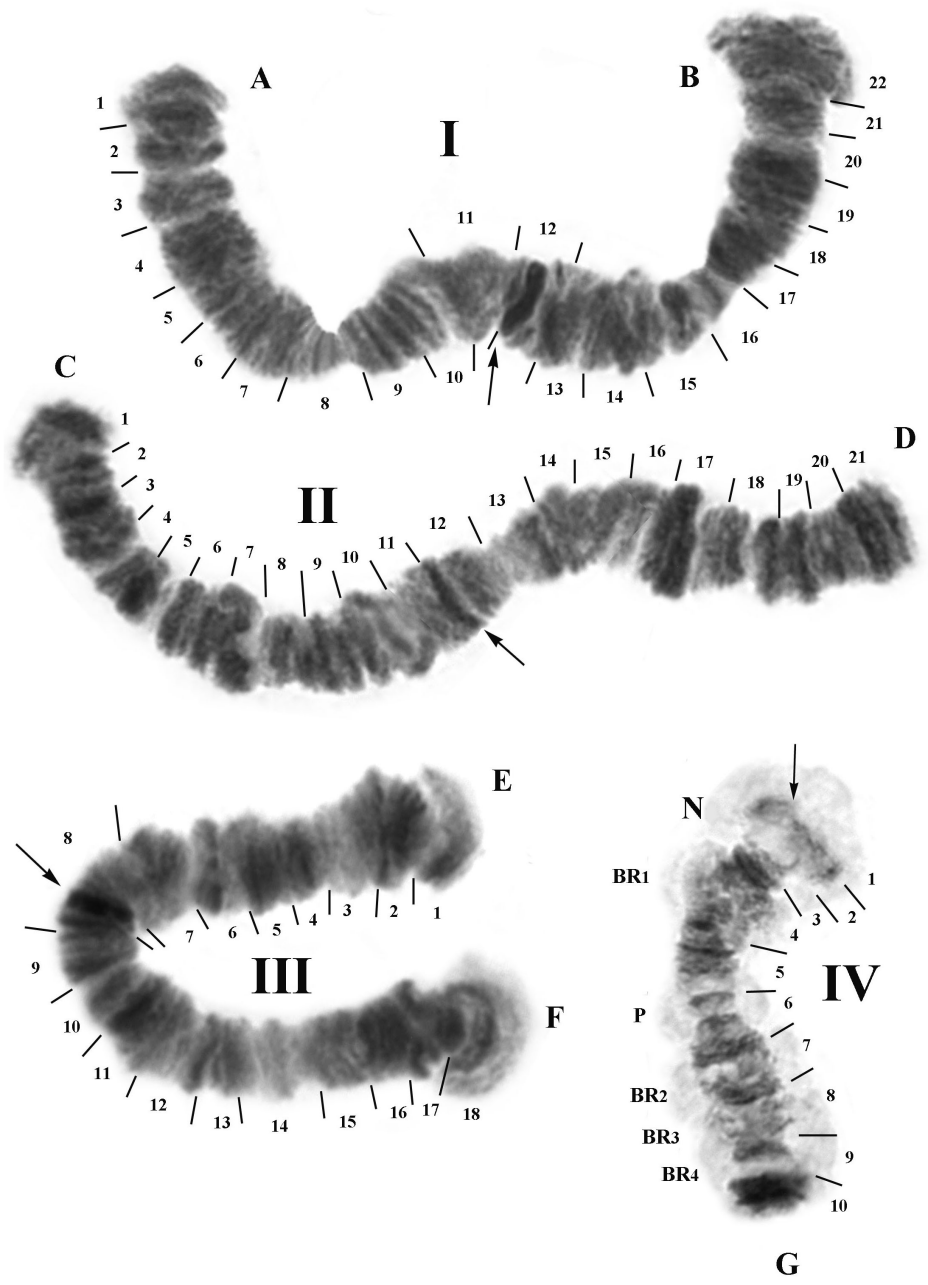
Representative karyotype of the population from Malawi. Chromosome numbers are indicated as **I, II, III** and **IV.** Chromosome arms are labeled **A–B, C–D, E–F**, and **G.** The expected locations of the centromeres are indicated by arrows and each section is numbered and delimited by short lines. N: nucleolus. BR_1_, BR_2_, BR_3_, BR_4_: Balbiani rings, P: puff.

As a whole, the morphology of the salivary glands was similar to those from the Nigerian population. They also contained 16–20 cells. However, the distinction between populations of cells with different sizes was not so obvious. The diploid chromosome number was also 2n=8 (Fig. 4). The combination of chromosomal arms was AB, CD, EF and G. Chromosomes were denominated from their respective lengths: I=II>III>IV. Chromosomes I and II were metacentrics, chromosome III submetacentric, and chromosome IV telocentric. Putative centromeres appeared as distinct dark heterochromatic blocks. The karyotype was mononucleolar: one obligatory nucleolus (N) was well developed in sec. 3, near the centromere and the telomere in arm G. Apart from the nucleolus N, four Balbiani rings (BR) were localized in chromosome IV. BR_1_ was close to N in sec. 4, and the three other BR followed one after another: BR_2_ in sec. 7, BR_3_ in sec. 8, BR_4_ in sec.9.

Chromosome I was arbitrarily divided into 22 sites. The putative centromere was localized in sec. 12 (Fig. 4). The narrowing in sec. 8 and also a group of five dark distinct bands in sec. 9–10 constituted Characteristic markers on the arm A. The arm B was easily distinguished due to the block of almost identical dark bands in sec. 19–20, and the narrowing in sec. 16.

Chromosome II was divided into 21 sections. The putative centromere was located in sec. 12 (Fig. 4). The arm C was easily distinguished due to the light area (sec. 5) bordered on both sides with groups of dark bands (sec. 5–6). In the arm D, we observed a narrowing in sec. 13 near the centromere, and groups of dark blocks in sec. 17–19, including the largest block of the karyotype in sec. 17.

Chromosome III was divided into 18 sections (Fig. 4). The putative centromere was localized in sec. 8. Both telomeres were fanlike. In both arms of E and F, dark blocks containing 4–5 bands were localized near the telomere (sec. 2. and sec. 16–17). These groups were separated from the telomere by conspicuous constrictions. A dense dark band was located between the centromere and sec. 9 in the arm F.

**Figure 5.**
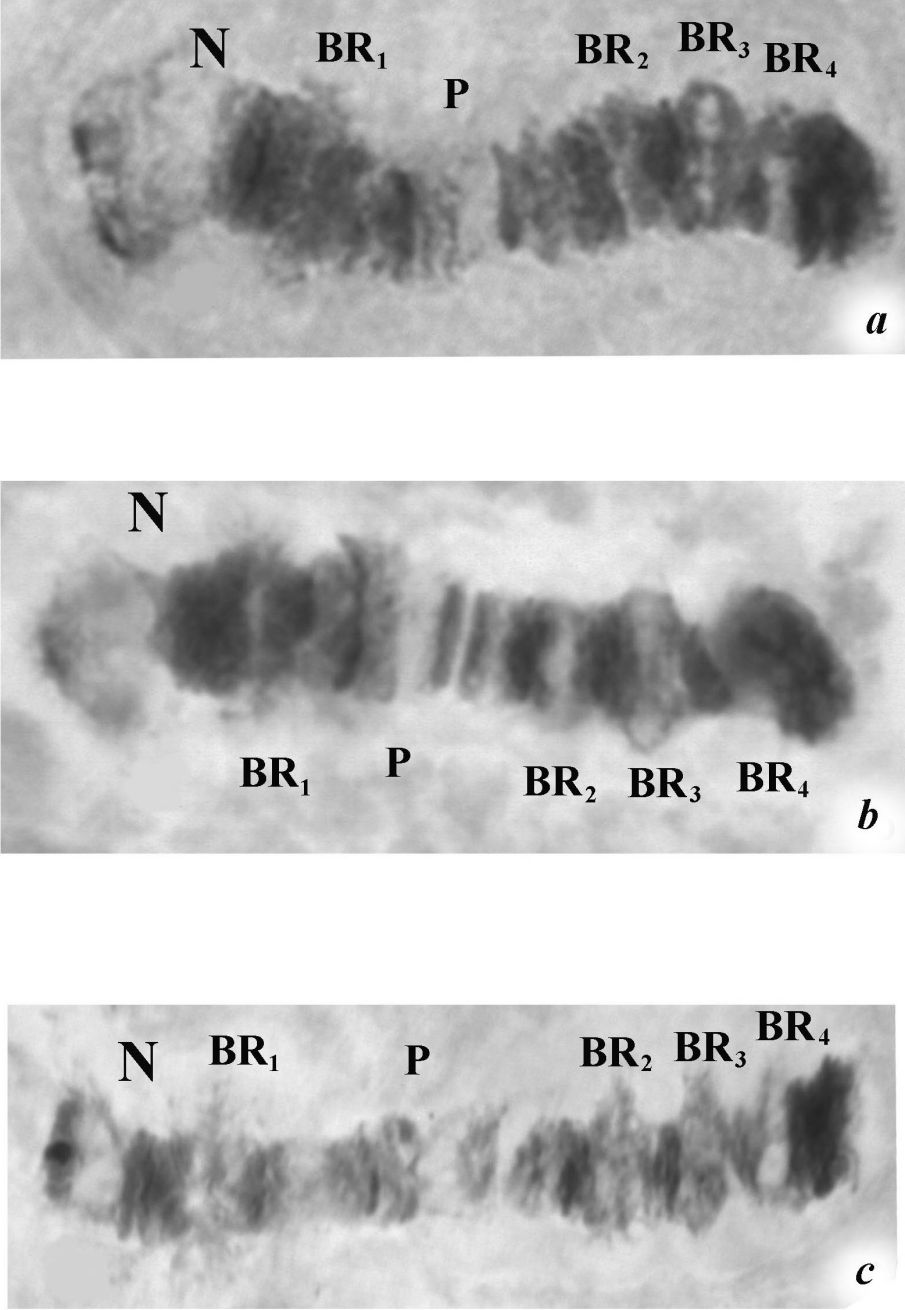
Chromosome IV patterns from different larvae of the Malawian population. Active regions show different levels of condensation. **a** N appears active, BR_1_, BR_2_, BR_3_, BR_4_ are slightly active **b** N and BR_3_ appear active, BR_1_ and BR_2_ are slightly active **c** N, BR_1_, BR_2_, BR_3_ and BR_4_ are active. N: nucleolus. BR_1_, BR_2_, BR_3_, BR_4_: Balbiani rings, P: puff.

Chromosome IV was divided into 10 sections and the putative centromere was located in sec. 1 (Fig. 4). In this chromosome were localized: the nucleolus N in sec. 3 and four Balbiani rings (BR), which considerably differed on their degree of activity. N and BR_1_ were constitutively active, while other BRs showed variable activity, with various combinations (Fig. 5).

Sometimes all BR were faintly active (Fig. 5a) and in other cases, BR_3_ showed a maximal activity (Fig. 5b). In the last case, BR_2_ and BR_3_ were expressed distinctly, whereas BR_4_ was only weakly expressed (Fig. 5c).

**Figure 6.**
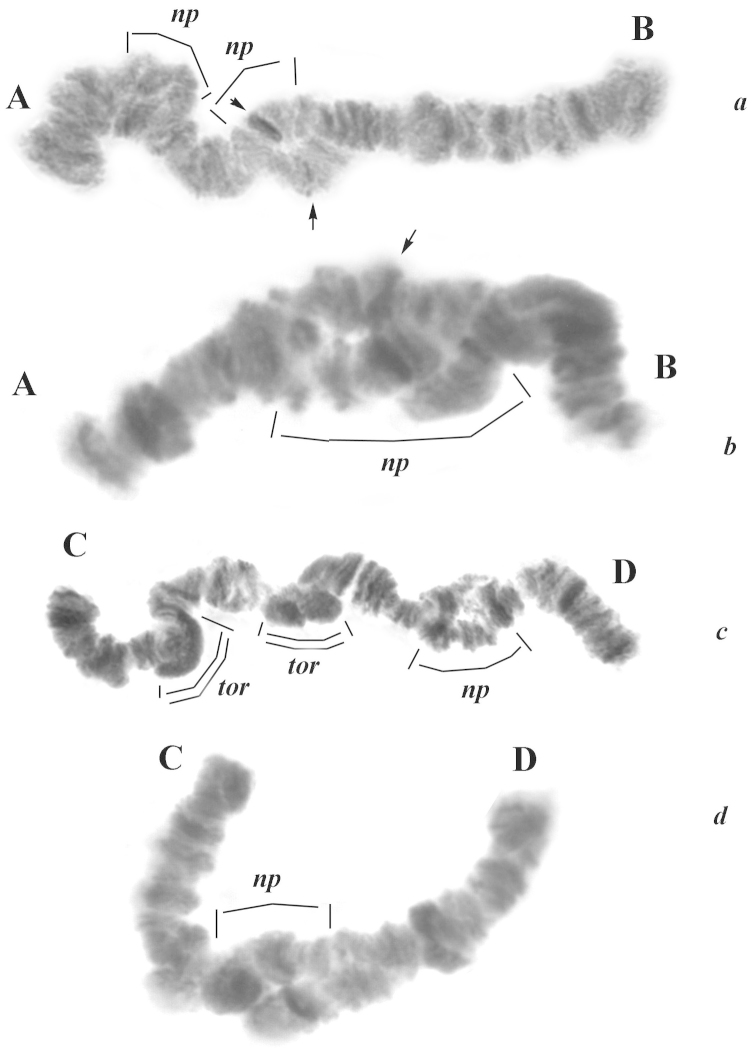
Different patterns of polymorphism for the chromosomes I and II in the Malawian population. **a** and **b** chromosome I. **c** and **d** chromosome II. Chromosome arms are labeled **A–B** and **C–D.** Arrows: putative centromeres, *np*: regions of non-pairing, *tor*: heterochromatic knots due to chromosome torsion.

Chromosomal polymorphism: For the majority of the studied individuals, we observed mispairing of the homologues. Uncoupled chromosome portions, as a result of torsion, were forming various structures. For example in the AB chromosome (Fig. 6a, b), homologous sections near the centromere were often situated nearby each other, due to asynapsis, and thus the area near the centromere appeared as a thickening.

Sometimes the regions of non-pairing (np) due to heterozygous inversions in the chromosome I (AB) were restricted to some bands only (Fig. 6a). Thus, the band of the centromere in one homologue was normally condensed. When the mispaired chromosome region was more extended, the band corresponding to the putative centromere was almost indistinguishable (Fig. 6b). The accurate band sequence in the arm A, close to the telomere on sec. 17–20 was broken too, due to non-pairing of the homologues and torsion of chromosomes. However, the non-pairing was sometimes observed throughout a large portion in the middle of the chromosome (Fig. 6b). In the chromosome II (arms CD), regions of non-pairing and torsions appeared as large heterochromatic knots (Fig. 6c, double lines). In this chromosome, extended non-pairing near the centromere was also observed quite often (Fig. 6d). Inversion polymorphism was not observed in this population.

### Karyotypical distinctions between the two populations

Karyotypical comparison between the two populations of *Polypedilum vanderplanki* showed considerable inter-population differences. The diploid number of chromosomes was identical (2n=8), but the band sequences on the chromosomes and the organization of active regions were substantially different. Whereas there was only one N in the Malawian population, the Nigerian population showed two major N. No similarity in the arrangement of the marker groups of bands was found in the long chromosomes. The differences in the morphology of the chromosomes IV were especially noticeable: in the Nigerian population, this chromosome showed one N_1_ and one BR, whereas one N and four BR were active in the Malawian population. In both populations, the fact that nucleoli and Balbiani Rings were active or not, should be related to the physiological status of the larva before fixation. Six days after rehydration, the influence of anhydrobiosis was probably negligible, but some larvae may have been engaged in the processes of metamorphosis, which could influence greatly the aspect of chromosomes by activating different transcriptional regions of the genome. However, the relative positions of nucleoli and Balbiani Rings on the chromosomes were completely different between the two populations and this may thus result most probably from populational variation, rather than from differences in the physiological or developmental status.

The major unique feature in the Nigerian *Polypedilum vanderplanki* karyotype was the low degree of polytenization of chromosome IV: it was only half as thick as the other chromosomes of the karyotype. Finally, the large heterozygous inversion present in the arm F of chromosome III was observed with a frequency of 100% in the Nigerian population, whereas the Malawian population did not show this chromosome rearrangement in the arm F.

It should be noted that the Nigerian population of *Polypedilum vanderplanki* is a highly inbred strain and this could explain the stability of its chromosomal rearrangement. In comparison, the Malawian population was a natural one, not inbred in the laboratory. Strong heterozygosity in this population could induce the mispairings, non-pairings and torsions, which were observed, especially on chromosomes I and II. In addition, desiccation-rehydration cycles are known to induce severe lesions to DNA and subsequent repair (Gusev et al. 2010). When larvae experience anhydrobiosis, chromosome morphology is thus likely to be affected. However, the karyotype pattern in the Nigerian population of *Polypedilum vanderplanki* was very stable and constant, even after rehydration. The detailed effect of anhydrobiosis on chromosome morphology is thus an issue that remains to be addressed. In addition to these important differences in bands pattern and general organization of active regions between both Nigerian and Malawian populations, similarities in the organization of the chromosomes were hardly observed in comparison to other published karyotypes in the genus *Polypedilum* (Porter and Martin 1977, Kerkis et al. 1996). The genus *Polypedilum* is actually very diverse with 8 subgenera described (Saether et al. 2010) and this could explain the poor similarity observed in the karyotypes between species spread over these subgenera.

To conclude, the karyotype and precise chromosome map of *Polypedilum vanderplanki* were determined for the first time and these data will be useful for future physical mapping of the genome data on the chromosomes. Besides, analysis of the karyotypes of Nigerian and Malawian samples showed important differences between both populations. Whereas chromosomal numbers were identical (2n=8), the morphology of chromosomes was totally divergent. Such important differences between populations exceed physiological variation and intraspecific polymorphism and to our point of view, these *Polypedilum* populations from Nigeria and Malawi should be considered as distinct species. The Nigerian population was originally collected in the Northern part of Nigeria, close to the locality where the type specimen used for the description of *Polypedilum vanderplanki* by Hinton (1951) was discovered. In addition, DNA sequence data showed that our laboratory Nigerian strain and Hinton’s samples were identical. Consequently, the karyotype presented here for the Nigerian population should be considered as the reference karyotype for *Polypedilum vanderplanki*. As a consequence, the Malawian population probably constitutes a new species of anhydrobiotic *Polypedilum*. Since examples of karyological studies on African chironomids are still scarce, accurate morphological description and physiological characterization of this new species of anhydrobiotic *Polypedilum* will be needed in the future.
